# PssP2 Is a Polysaccharide Co-Polymerase Involved in Exopolysaccharide Chain-Length Determination in *Rhizobium leguminosarum*

**DOI:** 10.1371/journal.pone.0109106

**Published:** 2014-09-30

**Authors:** Małgorzata Marczak, Paulina Matysiak, Jolanta Kutkowska, Anna Skorupska

**Affiliations:** 1 Department of Genetics and Microbiology, Institute of Microbiology and Biotechnology, Maria Curie-Skłodowska University, Lublin, Poland; 2 Chair and Department of Forensic Medicine, II Faculty of Medicine with English Language Division, Medical University, Lublin, Poland; University of Helsinki, Finland

## Abstract

Production of extracellular polysaccharides is a complex process engaging proteins localized in different subcellular compartments, yet communicating with each other or even directly interacting in multicomponent complexes. Proteins involved in polymerization and transport of exopolysaccharide (EPS) in *Rhizobium leguminosarum* are encoded within the chromosomal Pss-I cluster. However, genes implicated in polysaccharide synthesis are common in rhizobia, with several homologues of *pss* genes identified in other regions of the *R. leguminosarum* genome. One such region is chromosomally located Pss-II encoding proteins homologous to known components of the Wzx/Wzy-dependent polysaccharide synthesis and transport systems. The *pssP2* gene encodes a protein similar to polysaccharide co-polymerases involved in determination of the length of polysaccharide chains in capsule and O-antigen biosynthesis. In this work, a mutant with a disrupted *pssP2* gene was constructed and its capabilities to produce EPS and enter into a symbiotic relationship with clover were studied. The *pssP2* mutant, while not altered in lipopolysaccharide (LPS), displayed changes in molecular mass distribution profile of EPS. Lack of the full-length PssP2 protein resulted in a reduction of high molecular weight EPS, yet polymerized to a longer length than in the RtTA1 wild type. The mutant strain was also more efficient in symbiotic performance. The functional interrelation between PssP2 and proteins encoded within the Pss-I region was further supported by data from bacterial two-hybrid assays providing evidence for PssP2 interactions with PssT polymerase, as well as glycosyltransferase PssC. A possible role for PssP2 in a complex involved in EPS chain-length determination is discussed.

## Introduction

Polysaccharides are abundant components of bacterial cells as well as the matrices that they form in their ecological niches. Exopolysaccharides (EPS) are extracellular polysaccharides secreted by many bacteria that play several physiological roles. EPS produced by rhizobia protects bacteria from adverse conditions in the demanding environment of soil and are among the most important factors determining a successful symbiotic interaction between rhizobia and leguminous plants [1], [2]. Rhizobia living in the rhizosphere attach to the plant roots, invade plant tissues and colonize cells of the forming nodule, where they differentiate into bacteroids which provide fixed nitrogen for the plant in exchange for carbon [3]. Polysaccharide synthesis, independent of the glycoform produced, is a multistep process that employs several enzymatic and structural proteins. Generally, polysaccharides may be completely assembled in the cytoplasm before being targeted to the final location, such as the extracellular medium. Alternatively, they can be assembled in a form of repeating units, which are subsequently polymerized in the periplasm and transported to the external environment. Type 1 and 4 capsular polysaccharides of *Escherichia coli*, EPS and most O-antigens of Gram-negative bacteria follow the second pathway, i.e. assembly of the repeating unit in the cytoplasm and polymerization thereof in the periplasm combined with translocation outside the cell. This pathway is called the Wzx/Wzy-dependent pathway, as it requires the Wzx flippase and Wzy polymerase, unlike systems involving ABC-transporters or synthase proteins [4], [5].

Polysaccharide biosynthesis is initiated by glycosyltransferases involved in the assembly of the repeating unit on the lipid carrier undecaprenyl pyrophosphate. Complete subunits are then translocated to the periplasmic face of the inner membrane by the Wzx flippase [6], [7] and then polymerized by the Wzy polymerase to the extent that is regulated by the Wzc co-polymerase (Wzz in the case of LPS O-antigens) [8]. The nascent chain is translocated outside the cell by the Wza oligomeric channel protein [9]–[11]. Several genetic and structural studies revealed complex interrelations between proteins engaged in these processes, e.g. Wzx-Wzy/Wzz [12], Wzy-Wzz [13], [14], Wzc-Wza [15], as well as between glycosyltransferases [16], [17].

**Figure 1:**
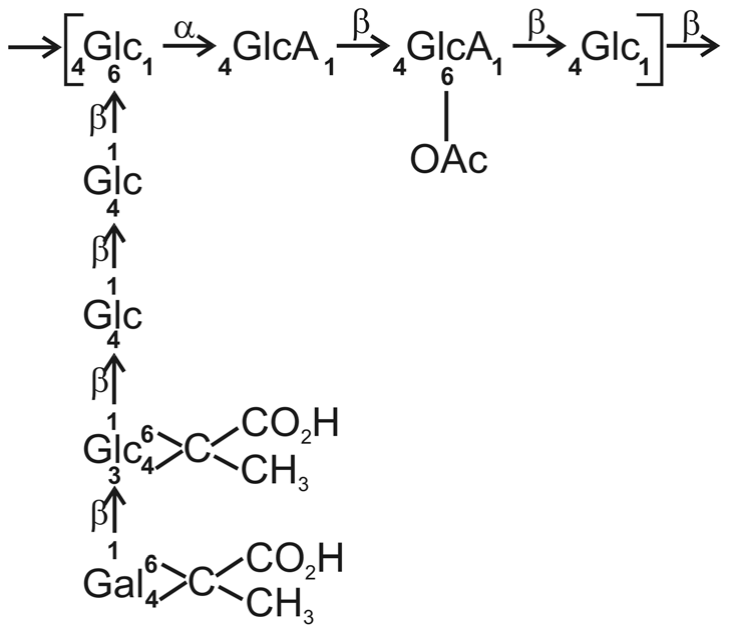
The chemical structure of the repeating unit of EPS produced by *R. leguminosarum* bv. *trifolii* [90]. Abbreviations: Glc, glucose; GlcA, glucuronic acid; Gal, galactose; Ac, acetyl.

Proteins engaged in EPS synthesis in *R. leguminosarum* bv. *trifolii* are encoded within the chromosomal Pss-I region. The region comprises genes encoding glycosyltransferases [18], [19], a putative flippase [20], a polymerase [21], a co-polymerase [22], and an outer membrane channel protein [23], [24]. The functions of several (but not all) glycosyltransferases encoded within the region were previously dissected. Glucosyl-IP-transferase PssA is the priming glycosyltransferase initiating the assembly of the octasaccharide EPS unit (**Figure 1**) by the transfer of UDP-glucose to the undecaprenyl phosphate lipid carrier attached to the cytoplasmic membrane [19], [25]. In the subsequent step, a glucuronosyl-(β-1,4)-glucosyl transferase composed of PssD and PssE catalyses the addition of a glucuronic acid residue [18], [26]. The addition of the second glucuronic acid is mediated by the glucuronosyl-β-1,4-glucuronosyltransferase PssC [18], [25], [26]. The outcome of mutations in *pssA*, *pssD*, *pssE*, and *pssC* is pleiotropic and in addition to abolishing the capacity to synthesize EPS, it affects the level of synthesis of several cellular proteins [27]. PssL is homologous to Wzx and was proposed to function as a flippase that translocates EPS subunits to the outer leaflet of the inner membrane [20]. PssT is homologous to Wzy and serves as a polymerase of EPS subunits; the *pssT* mutant produced EPS with a greater amount of high-molecular-weight (HMW) EPS than the wild type [21]. Polymerization of polysaccharides is influenced by a protein assigned to a family of polysaccharide co-polymerases (PCP) [28] that are distinguished by their common membrane topology with a large periplasmic loop flanked by two transmembrane segments [29]. PssP was demonstrated to be a PCP protein. It is a large inner membrane protein comprising a periplasmic domain with coiled-coils and two transmembrane segments [22], [30]. It was shown to be indispensable for EPS synthesis [22]. The terminal stage in the assembly of EPS, i.e. the translocation of a polymer across the outer membrane, occurs through the pore formed by the PssN lipoprotein homologous to Wza protein [23]. Bacterial two-hybrid (BTH) assays provided evidence for interactions between proteins involved in EPS biosynthesis and transport, namely PssP-PssT and PssP-PssN, consistent with the notion of a multicomponent complex [30].

Pss-I is likely not the only gene cluster involved in polysaccharide synthesis in *R. leguminosarum* RtTA1; several other regions with candidate genes were identified both in the chromosome and on a plasmid [31]. One of them, the chromosomal Pss-II region (GenBank Accession No. DQ384109), is comprised of several genes encoding putative homologues to constituents of the Wzx/Wzy pathway, suggesting involvement in the synthesis of EPS or other polysaccharide(s). One of the proteins encoded within the Pss-II cluster is PssP2. Its primary and predicted secondary structure similarity, protein topology, and subcellular localization resembled PCP proteins and indicated a possibility of PssP2 engagement in the synthesis of LPS and/or EPS.

To examine PssP2 involvement in the synthesis of either EPS or LPS, a mutant disrupted in the *pssP2* gene was constructed. The significance of this qualitative change in the PssP2 protein for production of EPS and LPS and the symbiotic phenotype was studied. Moreover, the interrelations between PssP2 and thus far characterized Pss proteins were also examined. The results obtained indicate that the PssP2 protein is yet another component of the protein complex that plays an important role in EPS chain-length determination.

## Materials and Methods

### Bacterial strains and culture conditions

Strains used in this work are listed in **Table 1**. *Escherichia coli* strains of general use were grown in lysogeny broth (LB) medium at 37°C [32], and *Rhizobium* strains were grown in TY [32], M1 with 1% glycerol [32] or 79CA with 1% mannitol or 1% glycerol at 28°C [33]. Bacterial two-hybrid (BTH) complementation assays were performed with the *E. coli cya* strain DHM1, which was grown at 30°C. Antibiotics were used at following final concentrations: ampicillin 100 µg/ml, kanamycin 40 µg/ml, gentamycin 5 (*E. coli*) or 10 µg/ml (*Rhizobium*), tetracycline 10 µg/ml and rifampin 40 µg/ml.

**Table 1:** Strains, plasmids and oligonucleotide primers used in this work.

Strain, plasmid or primer	Relevant description	Source or reference
***Escherichia coli***		
JM101	Δ*lac proAB thi supE* F′ *traD*36 *proAB lacI* ^q^ ZΔM15	[32]
S17-1	294 derivative RP4-2-Tc::Mu-Km::Tn*7* chromosomally integrated	[37]
DH5α	*supE*44 Δ*lacU*169 [Δ80 *lacZ*ΔM15) *hsdR*17 *recA*1 *endA*1 *gyrA*96 *thi*-1 *relA*1	[32]
DHM1	Reporter strain for BTH system; F^−^ *glnV*44(AS) *recA*1 *endA gyrA*96 *thi*-1 *hsdR*17 *spoT*1 *rfbD*1 *cya*-854	[62]
***Rhizobium leguminosarum*** ** bv. ** ***trifolii***		
RtTA1	Wild type strain, Str^r^, Rif^r^	[85]
RtP2-1.1	RtTA1, *pssP*::pKP2	This work
RtP2-1.1/P2his	*pssP*::pKP2 carrying pQBP2his plasmid	This work
**Plasmids**		
pK19mobGII	pUC19 derivative, *lacZ*, *mob, gusA*; Km^r^	[34]
pKP2	pK19mobGII with 482-bp PstI-SalI fragment of *pssP2* gene	This work
pMP220	IncP, *mob*, promoterless *lacZ*, Tc^r^	[35]
pMPO1	pMP220 with 681-bp *Eco*RI fragment carrying *pssO* promoter	[38]
pMP2P	pMP220 with 1.1-kb BamHI-PstI fragment of pARF136 carrying the *pssP* promoter	[22]
pMP-P2	pMP220 with 245-bp BglII-XhoI fragment carrying putative *pssP2* promoter	This work
pMP-Y	pMP220 with 424-bp BglII-XhoI fragment carrying putative *pssY* promoter	This work
pUT18	Two-hybrid plasmid for *cyaA*T18 fusion construction, Amp^r^	[62]
pUT18C	Two-hybrid plasmid for *cyaA*T18 fusion construction, Amp^r^	[62]
pKT25	Two-hybrid plasmid for *cyaA*T25 fusion construction, Km^r^	[62]
pUT18C-zip	Two-hybrid control plasmid	[62]
pKT25-zip	Two-hybrid control plasmid	[62]
*pssP/pssT/pssL*pUT18	Two-hybrid plasmid containing *cyaA*T18-*pssP*, *pssT*, *pssL* fusion, respectively	[30]
*pUT18CpssP/pssT/pssL*	Two-hybrid plasmid containing *cyaA*T18-*pssP*, *pssT*, *pssL* fusion, respectively	[30]
*pKT25pssP/pssT/pssL*	Two-hybrid plasmid containing *cyaA*T25- *pssP*, *pssT*, *pssL* fusion, respectively	[30]
*pssP2/pssA/pssC*pUT18	Two-hybrid plasmid containing *cyaA*T18-*pssP2*, *pssA*, *pssC* fusion, respectively	This work
*pUT18CpssP2/pssA/pssC*	Two-hybrid plasmid containing *cyaA*T18-*pssP2*, *pssA*, *pssC* fusion, respectively	This work
*pKT25pssP2/pssA/pssC*	Two-hybrid plasmid containing *cyaA*T25- *pssP2*, *pssA*, *pssC* fusion, respectively	This work
pQE30	Expression vector, Amp^r^	Qiagen
pBBR1MCS-5	*mob* Gm^r^	[36]
pQE30/P2his	pQE30 with *pssP2* gene devoid of its own ATG and STOP codons cloned into SacI-HindIII	This work
pQBP2his	pBBR1MCS-5 with expression cassette from pQE30P2his cloned into XhoI-HindIII	This work
**Primers**		
CFwBTH	5′AAA**TCTAGA**TAATCAGCAAAAGACTTTTCCGCAT3′	amplification of the *pssC* gene for the BTH system (XbaI recognition site)
CRvBTH	5′AAA**GGATCC**TGGGCGGCATTGGGTTTGTATTC3′	amplification of the *pssC* gene for the BTH system (BamHI recognition site)
AFwBTH	5′AAA**GTCGAC**AGGGTTAACCATTGATCGCCTATTGC3′	amplification of the *pssA* gene for the BTH system (SalI recognition site)
ARvBTH	5′AAA**GGATCC**AAGCCTTTACCACCGGTCAGCTCCGAC3′	amplification of the *pssA* gene for the BTH system (BamHI recognition site)
AFwBTH2	5′AAA**TCTAGA**GACAGGGTTAACCATTGATCGCCTA3′	amplification of the *pssA* gene for the BTH system (XbaI recognition site)
P2FwBTH	5′AAA**TCTAGA**GACCTCAAGCACGATCTTCAGCGGTGT3′	amplification of the *pssP2* gene for the BTH system (XbaI recognition site)
P2RvBTH	5′AAA**GGTACC**CCTGACTCTATTCTTTTCGGTGCATGAT3′	amplification of the *pssP2* gene for the BTH system (BamHI recognition site)
P2exFWSacI	5′AAA**GAGCTC**ACCTCAAGCACGATCTTCAGCG3′	amplification of *pssP2* gene for cloning into the expression vector (SacI recognition site)
P2exRVHindIII	5′AAA**AAGCTT**TGACTCTATTCTTTTCGGTGC3′	amplification of *pssP2* gene for cloning into the expression vector (HindIII recognition site)
P2prom_fw	5′AAA**AGATCT**ACGATGTCAGTTATGAGTACC3′	amplification of putative *pssP2* promoter (BglII recognition site)
P2prom_rv	5′AAA**CTGCAG**GTCGTCCTAATCCAAAATGGC3′	amplification of putative *pssP2* promoter (PstI recognition site)
Yprom_fw	5′AAA**AGATCT**TTATATTGGTCTTAATATGAG3′	amplification of putative *pssY* promoter (BglII recognition site)
Yprom_rv	5′AAA**CTGCAG**CGGTGGTCTCCAAAATATTC3′	amplification of putative *pssY* promoter (PstI recognition site)
pUCfw	5′CCCAGTCACGAAGTTGTAAAACG3′	universal primer used for checking the type of genomic rearrangements in the *pssP2::*pKP2
pUCrv	5′AGCGGATAACAATTTCACACAGG3′	universal primer used for checking the type of genomic rearrangements in the *pssP2::*pKP2

Oligonucleotides were purchased from Genomed (Warsaw, Poland). Abbreviations: Str^r^, streptomycin resistance; Rif^r^, rifampin resistance; Km^r^, kanamycin resistance; Tc^r^, tetracycline resistance; Amp^r^, ampicillin resistance; Gm^r^, gentamicin resistance.

### Plasmid constructions for mutagenesis, promoter probing and *pssP2* overexpression

Plasmids and primers used in this work are listed in **Table 1**. Standard protocols for genomic DNA isolation, PCR, molecular cloning, transformation and DNA analysis were used [32]. pKP2 plasmid used for integration mutagenesis of *pssP2* gene was constructed by subcloning of the PstI-SalI fragment of *pssP2* gene into the pK19mobGII vector [34]. Promoter probe constructs pMP-P2 and pMP-Y resulted from cloning of PCR products covering putative *pssP2* and *pssY* promoters into the BglII-PstI restriction sites of pMP220 vector [35]. pQBP2his plasmid encoding a His-tagged version of PssP2 was constructed using pQE30 expression vector (QIAGEN) and pBBR1MCS-5 [36]. For that purpose, *pssP2* was amplified with Pfu polymerase (Thermo Scientific) using P2exFWSacI and P2exRVHindIII primers. The amplicon was cloned between the SacI and HindIII restriction sites in the pQE30 expression vector and used to transform *E. coli* JM101. The entire His_6_-*pssP2* expression cassette that comprised: the *pssP2* ORF with the His_6_-tag in the corresponding reading frame, as well as the promoter and the operator sequences, was subcloned into XhoI-HindIII of pBBR1MCS-5, which is a broad host range vector able to replicate in *Rhizobium*.

### Construction of RtTA1 chromosomal insertion mutant *pssP2*::pKP2

pKP2 plasmid was transferred from *E. coli* S17-1 to RtTA1 by conjugation and transconjugants were selected on 79CA medium with kanamycin. Bacterial mating experiments were performed as described by Simon et al. [37]. The clones with pK19mobGII chromosomal integration were selected on 79CA medium supplemented with 5-bromo-4-chloro-3-indolyl-β-D-glucuronide substrate for β-glucuronidase (GUS). The selected kanamycin-resistant and GUS^+^ clone was probed for genomic rearrangements by PCR with primers matching the *pssP2* gene and pUC universal primers matching vector sequences.

### Promoter activity assays

pMP-P2 and pMP-Y plasmids carrying putative promoters of *pssP2* and *pssY* genes were introduced into RtTA1 by electroporation. Activities of promoters were measured in cultures of respective strains grown in TY, 79CA with 1% mannitol or M1 with 1% glycerol, as the activity of β-galactosidase reporter enzyme and expressed in Miller units. The assay was performed in single tubes format. pMPO1 plasmid carrying the *pssO* gene promoter [38] served as positive control and pMP2P plasmid carrying the *pssP* gene promoter [22] served as the reference for the activity of the gene encoding a PCP protein. The empty pMP220 vector served as a negative control.

### Co-purification of interacting proteins

The level of His_6_-PssP2 production in *E. coli* JM101 carrying pQBP2his was very low and the protein was difficult to identify through Western blotting with anti-His antibodies. pQBP2his plasmid was introduced to the RtP2-1.1 integration mutant by electroporation. Both the mutant and its complemented derivative were cultivated for 3 days in 100 ml of 79CA medium with kanamycin and gentamycin where appropriate. Then, the cells were harvested by centrifugation at 6000× *g* at 4°C, washed and subjected to *in vivo* cross-linking with 0.5% formaldehyde according to the procedures described previously [23], [30]. Afterwards the cells were washed and resuspended in 50 mM sodium phosphate, 300 mM sodium chloride buffer, pH 7.0 and disrupted in a French press (one passage at 18,000 psi). Crude lysate was clarified to remove unbroken cells and debris by centrifugation at 6000× *g* at 4°C and the supernatant was ultracentrifuged at 85,000× *g* for 1 h to sediment the membranes. The membranes were resuspended in 50 mM sodium phosphate, 300 mM sodium chloride buffer, pH 7.0 and supplemented with DDM (n-dodecyl-beta-D-maltoside; Sigma) to a final concentration of 0.2%. Membrane proteins were solubilized in a cold room for 1 h. Then, the mixture was centrifuged at 14,000× *g* for 30 min, the supernatant was collected and mixed with TALON metal affinity His-select resin (Clontech). Mixing was performed at 4°C overnight. After mixing, the resin was collected by centrifugation for 2 min at 700× *g* and 10 volumes of binding/wash buffer (50 mM sodium phosphate, 300 mM sodium chloride buffer, pH 7.0) was added and mixed on ice for 10 min. This wash step was repeated four times before the final elution step. The last washing contained 5 mM imidazole. Relevant protein(s) were eluted using 50 mM sodium phosphate, 300 mM NaCl, 150 mM imidazole buffer, pH 7.0. Samples were collected and analyzed by SDS-PAGE and Western immunoblotting with anti-His and anti-PssP antibodies.

### Plant tests

Red clover (*T. pratense* L. cv. Rozeta) seeds were surface sterilized, germinated, and grown as described previously [39]. Four-day-old clover seedlings were planted in sterile nitrogen-free slants (one per tube) and allowed to grow for 4 days before inoculation with 0.2 ml of cell suspension at an approximate density of 1.0×10^9^ cells/ml. Five weeks after the inoculation, plants were harvested and examined for nodulation and nitrogen fixation phenotype. Nitrogen fixation was evaluated indirectly, on the basis of color and green matter production, which was estimated by weighing the shoots. Twenty clover plants were used for each strain.

### Cell-surface polysaccharide analysis

Extracellular exopolysaccharides were precipitated with 3 volumes of ethanol from the supernatants of bacterial cultures grown with shaking for 3 days in 100 ml of 79CA medium with 1% glycerol. EPS was fractionated twice on 0.7 cm×90 cm Sepharose CL-6B column (Sigma-Aldrich). Fractions of 1 ml were collected and the total sugar content was determined according to Yasar [40] and calculated in glucose equivalents. Obtained results were averaged. Molecular weight markers used were: Blue Dextran, 2 MDa; Dextran T550, 550 kDa, and Dextran T10, 10 kDa. The glycosyl composition analysis of EPSs were determined by preparation of the alditol acetate derivatives, identified and quantified by gas liquid chromatography mass spectrometry (GLC-MS).The samples were hydrolyzed (120°C, 2 h) in 2 M trifluoroacetic acid (TFA) reduced with sodium borodeuteride (NaBD_4_), and acetylated. To confirm the presence of uronic acids, methanolysis and carboxyl reduction with NaBD_4_ prior to TFA hydrolysis was performed [41]. LPS from *R. leguminosarum* strains was isolated by whole cell microextraction using proteinase K digestion as described by Apicella [42]. Electrophoresis was carried out on a 12.5% SDS-PAGE polyacrylamide gel using a tricine buffer system [43] and visualised by oxidative silver staining according to the method of Tsai and Frasch [44].

### Sedimentation/autoaggregation analysis

For sedimentation analyses the method described by Sorroche et al. [45], with minor modifications, was employed. *Rhizobium* strains were grown in 79CA medium with 1% mannitol at 28°C for 24 h, diluted to the same OD_600_ 0.1 and grown for 48 h. Afterwards, 5 ml of the cultures were transferred to agglutination tubes, measured for OD_600_ (A_0_) and then allowed to settle by incubation without agitation for 24 h at 4°C. Next, OD_600_ was measured for 0.3 ml of the upper layer of suspension (A_t_). The autoaggregation percentage was expressed as follows: 1−(A_t_/A_0_)×100 [45], averaged from five independent experiments and subjected to statistical analyses.

### Analyses of proteins

Proteins were analyzed by SDS-PAGE and either visualized by PageBlue Staining Solution (Thermo Scientific) or electroblotted onto PVDF membrane (Millipore). Immunoblots were probed with the primary: anti-PssP [30] and anti-His antibodies (Roche) and secondary anti-rabbit and anti-mouse IgG antibodies conjugated with alkaline phosphatase (Sigma).

### BTH testing

The ‘bait’/‘prey’ vectors pKT25, pUT18C and pUT18, and the control plasmids pKT25-zip and pUT18C-zip were used in protein interaction analyses. To construct plasmids encoding Pss-CyaA fusion proteins, *pssP2*, *pssA* and *pssC* genes were PCR amplified using appropriate primers listed in **Table 1**, with RtTA1 genomic DNA as the template. Amplified DNA fragments were digested with appropriate restriction enzymes (Thermo Scientific), the names of which are indicated alongside the names of primers used for amplification in **Table 1**, subcloned into the corresponding sites of the pKT25, pUT18C and pUT18 vectors and transformed into *E. coli* DH5α strain. Constructs were verified by sequencing. Plasmids were then transformed into *E. coli* DHM1 reporter strain, with selection on LB agar medium containing ampicillin, kanamycin, X-gal and IPTG. For a quantitative measurement of interaction strength β-galactosidase activity was measured as described previously [30]. The assay was performed in single tubes format. Construction of BTH plasmids with *pssT*, *pssP* and *pssL* genes was described previously [30].

### Bioinformatic analyses

Putative homologues to PssP2 were identified via BLASTp [46]. HHpred tool was used to search against existing structures of PCP proteins in the PDB (Protein Data Bank) [47]. For the alignment of PssP and PssP2 ClustalW [48] was used and the result was visualized with Alignment Viewer. HHpred and alignment tools are available at http://toolkit.tuebingen.mpg.de. Searching for tyrosine kinase motifs was assisted by BYKdb [49]. Protein subcellular localization was predicted with PSORTb [50]. Protein topology was analysed using: DAS [51], TMHMM [52], HMMTOP [53] and Phobius [54]. Coiled-coil regions were predicted with COILS [55] and NetPhos 2.0 server [56] was used to predict phosphorylation sites. Putative promoters were predicted with Neural Network Promoter Prediction [57] and Rho-independent terminators were searched for using ARNold tool [58].

### Statistical analyses

The results of promoter activity assays, plant tests, autoaggregation assays as well as bacterial two-hybrid results were submitted for statistical analyses, which were performed with STATISTICA software, using one-way analysis of variance (ANOVA) and the Tukey test at a significance level of p<0.05.

## Results

### PssP2 is a homologue of polysaccharide co-polymerases

**Figure 2:**
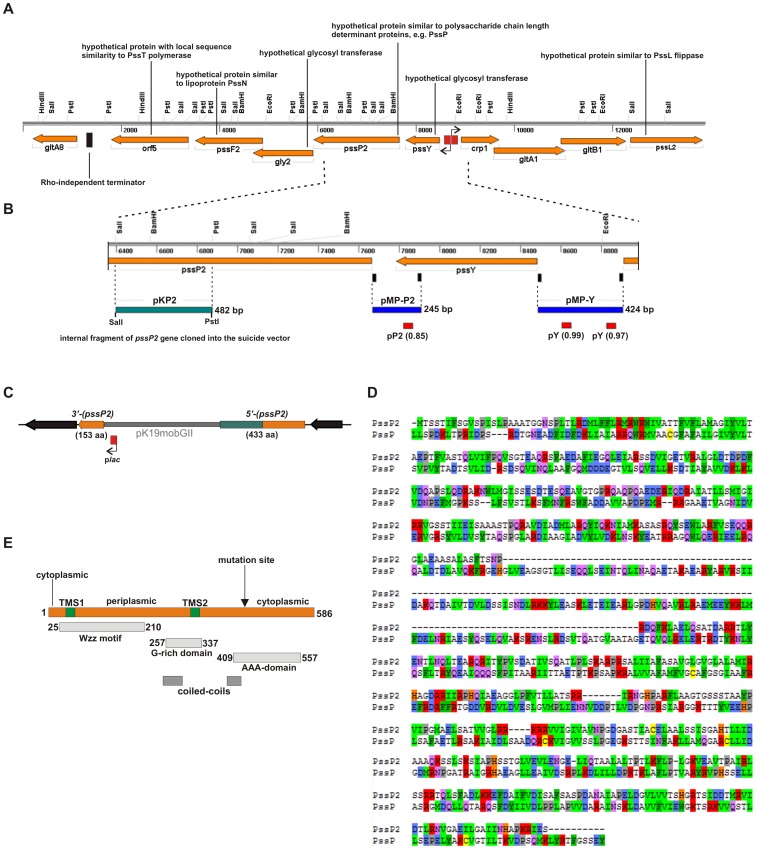
Organization of genes in the Pss-II region, constructs used in the *pssP2* gene functional analyses and the results of PssP2 protein amino acid sequence analyses. A) Physical and genetic map of the *R. leguminosarum* bv. *trifolii* Pss-II region; genes encoding putative proteins similar to elements of the Wzx/Wzy-dependent polysaccharide polymerization pathway are indicated above the map; Rho-independent terminator predicted downstream *orf5* gene is marked with a *black rectangle*, promoters predicted between the *pssY* and *pssP2* genes are marked with *red rectangles*; B) Constructs used for integration mutagenesis of the *pssP2* gene (pKP2; *green bar*) and probing putative promoters identified upstream the *pssP2* (pMP-P2) and *pssY* (pMP-Y) genes (*blue bars*). Small *black rectangles* mark positions of primers used for amplification of promoter regions. *Red rectangles* below the pMP-P2 and pMP-Y constructs mark positions of identified promoters and the scores obtained for each predicted promoter. C) Genomic organization of the integration mutant *pssP2*::pKP2. Position of the p*lac* promoter (*red rectangle*) in the vector part and the direction of transcription from the promoter are shown. D) Sequence alignment of PssP2 and PssP proteins of *R. leguminosarum* bv. *trifoli* TA1. The alignment was produced in ClustalW and visualised by Alignment Viewer; amino acids were coloured according to their biochemical properties, thus the same colour means either identity or similarity, e.g. positively charged amino acids Arg and Lys are marked in red. E) Scheme of PssP2 topology and specific motifs found *in silico*. Blocks representing domains are aligned respective to the location in the polypeptide; TMS, transmembrane segment.

The chromosomal Pss-II region in *R. leguminosarum* bv. *trifolii* TA1 (RtTA1) [31] is located ∼200 kb from Pss-I and comprises several genes encoding putative proteins similar to proteins engaged in the model Wzx/Wzy-dependent pathway of polysaccharide assembly, suggesting involvement in the synthesis of EPS or other polysaccharide(s) in this strain (**Figure 2A**). One of the genes in the region, *pssP2*, encodes a hypothetical protein (586 amino acids) similar to the PssP co-polymerase encoded within the Pss-I region. The local level of identity/similarity between PssP2 and PssP (scored with BLASTp) was shown to be 28%/47% in the region covering 258–554 aa of PssP2 and 27%/45% in the region covering 16–251 aa of PssP2 (**Table 2**). BLASTp searches throughout the UniProtKB/SwissProt database revealed similarity of PssP2 to known bacterial kinases involved in polysaccharide production (**Table 2**). BLASTp searches among non-redundant protein sequences revealed hits with prevalence of putative sugar transporter proteins as well as LPS and EPS biosynthesis proteins in *Rhizobia* (thus showing that the genes homologous to *pssP2* are common in this group of bacteria). The most similar sequences were found in *R. leguminosarum* and *R. etli* (the lowest identity/similarity ∼80%/90%), while the least similar sequences originated from the *R. phaseoli* species and genera *Mesorhizobium*, *Sinorhizobium* (*Ensifer*), and *Bradyrhizobium* (up to ∼30%/40% identity/similarity; all the sequences were similar in length).

**Table 2:** Putative homologues of PssP2 protein (586 aa) (ABD36550) identified through BLASTp searches.

Homologous protein (aa)	Bacterium	Identity/similarity (%) (query coverage)	Accession number	Function	Reference
ExoP (786)	*Sinorhizobium meliloti*	29/44 (207–578) 26/42 (38–251)	P33698	Succinoglycan biosynthesis transport protein ExoP	[86]
Wzc (720)	*Escherichia coli*	22/41 (232–583)	P76387	Tyrosine-protein kinase Wzc	[73]
CpsD (232)	*Streptococcus agalactiae*	29/43 (406–584)	Q3K0T0	Tyrosine-protein kinase CpsD	[87]
Ptk (733)	*Acinetobacter johnsonii*	23/39 (219–578)	O52788	Tyrosine-protein kinase Ptk	[88]
Etk (726)	*Escherichia coli*	21/35 (255–583) 26/43 (181–310)	P58764	Tyrosine-protein kinase Etk	[89]
PssP (746)	*Rhizobium leguminosarum* bv. *trifolii* TA1	28/47 (258–554) 27/45 (16–236)	ABD47316	Protein involved in EPS chain length determination	[22], [24]

The database used above was the non-redundant UniProtKB/SwissProt. The multiple alignment of the above mentioned sequences is presented in **Figure S1 (Supplementary data)**.

The hypothetical PssP2 amino acid sequence is characterized by the presence of several motifs: the Wzz motif (Pfam: PF02706) between 25–210 aa, found in chain length determinant proteins involved in lipopolysaccharide biosynthesis (PCP-1 family of proteins) as well as in bacterial tyrosine kinases (PCP-2 family of proteins), the G-rich domain (PF13807) on putative tyrosine kinase (between 257–337 aa), and the AAA-domain (ATPases Associated with diverse cellular Activities) (PF13614) between 409–557 aa (**Figure 2E**). Even though proteins similar to PssP2 and with an experimentally verified function are bacterial kinases, PssP2 probably does not have a kinase activity. BYKdb-assisted analysis (Bacterial tYrosine-Kinase database, which contains computer-annotated BY-kinase sequences) [49] of the PssP2 amino acid sequence revealed that it does not possess motifs specific for this group of proteins, i.e. the Walker A, A′, and B as well as the C-terminal Y-cluster, found in the model Wzc protein [59]. On the contrary, the PssP protein (Pss-I region) possesses all motifs specific for this class of proteins except for the C-terminal Y (tyrosine)-cluster [22]. NetPhos analysis revealed that PssP2 might be phosphorylated on serine (25 sites), threonine (7 sites), or tyrosine (2 sites).

PssP2 is much smaller than PssP (their lengths are 586 aa and 746 aa, respectively) (**Table 2**) but its topology resembles Wzc-like proteins, not Wzz-like proteins. The PssP2 periplasmic domain is much shorter than in PssP (alignment of the two sequences clearly shows that there is a huge gap in the periplasmic domain of PssP2) (**Figure 2D**), and contrary to Wzz proteins, PssP2 possesses a long cytoplasmic domain yet devoid of specific kinase motifs.

Similarly to its homologues, PssP2 was predicted to be an inner membrane-embedded protein (PSORTb) with two transmembrane regions located around amino acid positions 44–56 and 316–331 (DAS), 41–60 and 315–334 (TMHMM), 41–60 and 314–333 (HMMTOP), 39–58 and 314–334 (Phobius) (**Figure 2E**), and with both termini located in the cytoplasm. The PssP2 topology predicted by TMHMM is exceptional and indicates orientation of the termini to be ‘out-out’. PssP2 was also predicted to form coiled-coils in the periplasmic domain (namely one significant coil) and in the C-terminal region (one secondary coil predicted with lower confidence) (COILS) (**Figure 2E**).

Multiple sequence alignment of PssP2 and homologous proteins listed in **Table 2** (**Figure S1**) as well as search against protein structures deposited in PDB with HHpred tool (**Table 3**) revealed that the C-terminal domain of PssP2 may have a conserved fold resembling that of bacterial protein kinases. Moreover, despite the small number of published X-ray crystal structures of PCP periplasmic domains, the corresponding region of PssP2 was matched against PDB structures 4E29, 3B8O and 3B8M (**Table 3**). Together, these results provide several lines of evidence supporting homology of PssP2 to PCP proteins.

**Table 3:** Summary of the highest scoring results from the HHpred search of the PssP2 protein against the PDB database.

Match	Organism	Probability/e-value	Match region in query PssP2 (aa)	Match region in found template (aa)	Secondary structure score	PDB identifier
Tyrosine-protein kinase Etk	*Escherichia coli*	100.0/2.1e-38	328–586	13–282 (299)	26.7	3CIO
Tyrosine-protein kinase Wzc	*Escherichia coli*	100.0/1.7e-38	335–586	12–270 (286)	25.6	3LA6
Tyrosine-protein kinase CapA (C-terminal fragment)	*Staphylococcus aureus*	100.0/7.6e-36	337–587	13–258 (269)	27.8	4JMP
Cell division inhibitor ATPase MinD	*Pyrococcus furiosus*	99.9/2.2e-23	408–585	2–178 (237)	17.6	1G3Q
Bacterial cell division regulator MinD	*Archaeoglobus fulgidus*	99.9/8.1e-23	408–584	2–176 (263)	18.8	1HYQ
Chimeric WzzB chain length determinant protein (periplasmic domain)	*Shigella flexneri*	97.8/0.001	60–312	7–248 (248)	16.8	4E29
Lipopolysaccharide biosynthesis protein WzzE	*Escherichia coli*	97.3/0.0056	63–309	1–265 (265)	15.5	3B8O
Bacterial polysaccharide co-polymerase FepE	*Escherichia coli*	97.0/0.033	62–305	12–279 (280)	17.4	3B8M

### Transcriptional activity of the *pssP2* gene

Organization of genes in the Pss-II region, i.e. their orientation and spacing between putative ORFs, suggested that they formed two transcriptional units with promoters located between *pssY* encoding a putative glycosyltransferase and *crp1* and active in both directions (**Figure 2A**). Analysis of the 5′-upstream region of *pssP2* revealed that the gene might be preceded by a weak promoter (predicted with 0.85 score by Neural Network Promoter Prediction) localized in the 3′-end of the preceding *pssY* gene (**Figure 2B**). Stronger promoters were predicted to be localized upstream of the *pssY* gene (two promoters with the highest scores, 0.97 and 0.99, are marked in **Figure 2B**).

**Figure 3:**
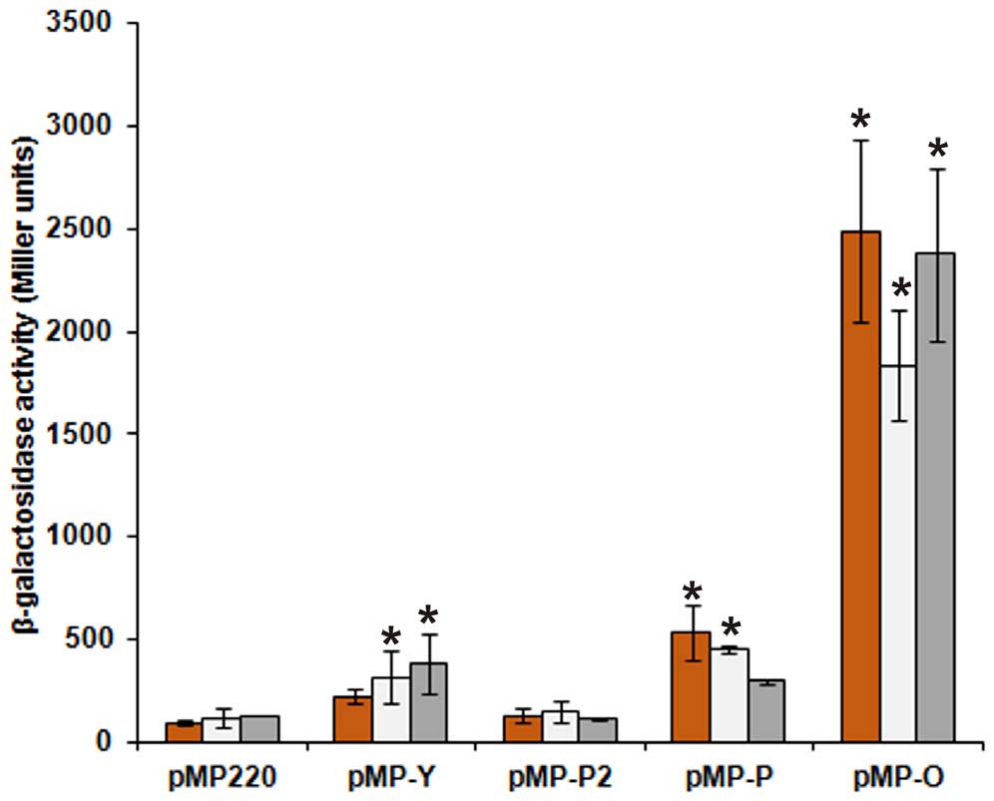
Transcriptional activity of the predicted *pssP2* and *pssY* promoters, as determined by measuring β-galactosidase activity in *R. leguminosarum* bv. *trifolii* TA1. The strains carrying a plasmid with appropriate promoter fusion were cultured in TY (*orange bars*), 79CA (*light grey*) or M1 (*dark grey*) medium. Values are the means ± standard errors (*extended bars*) of at least four independent assays and are expressed in Miller units. The bars labeled with asterisks represent β-galactosidase activity values which are significantly different from the empty pMP220 vector control at p<0.05. The results were compared within groups for the three different media. The original names of the constructs for *pssO* and *pssP* genes were changed to avoid confusion only for the sake of data presentation: pMP-P stands for the original pMP2P [22] and pMP-O stands for the pMPO1 [38].

To determine whether the predicted *pssP2* weak promoter is active in RtTA1, a DNA fragment covering this hypothetical promoter was PCR-amplified and cloned into pMP220 resulting in pMP-P2 (**Figure 2B** **,** **Table 1**). The level of β-galactosidase activity measured in RtTA1 was not different from that of the pMP220 vector alone (**Figure 3**). Taking into account lack of detectable activity, an additional fragment preceding the *pssY* gene was cloned into pMP220 to give pMP-Y (**Figure 2B** **,** **Table 1**) to assess whether there is a promoter that might drive the transcription of *pssY* and *pssP2*. In this case, the level of β-galactosidase activity was significantly higher than in the control in the case of cultures grown in 79CA and M1 media (**Figure 3**). For comparison, the strong promoter of the *pssO* gene [32] showed ∼20-fold increase in β-galactosidase activity in comparison with pMP220 regardless of the medium used (**Figure 3**). The promoter of the *pssP* gene [22], revealed significant increase in activity in case of cultures grown in TY and 79CA medium (**Figure 3**).

According to the results obtained, it is proposed that *pssP2* transcription is most probably driven by a medium active promoter that precedes *pssY* gene. In the light of the above data, lack of the strong RBS upstream of the *pssP2* ORF, the presence of several rare Arg codons in the 5′-end and the data for other genes implicated in the Wzx/Wzy-dependent pathway, the products of which are involved in polysaccharide polymerization and transport, it is predicted that the PssP2 protein may not be abundant in RtTA1 cells.

### PssP2 is involved in EPS synthesis

To investigate the function of *pssP2* and verify its involvement in polysaccharide synthesis, a mutant disrupted in the *pssP2* open reading frame through the integration of the plasmid was constructed. To this end, the integration plasmid pKP2 carrying an internal fragment of *pssP2* (**Figure 2B**) was introduced into the RtTA1 wild type and its integration was forced by an antibiotic selection. The resulting *pssP2*::pKP2 mutant was checked for the type of genomic rearrangements through PCR (data not shown). The results showed that the mutant encoded a shorter variant of PssP2 (N-terminal 433 aa) and the 3′-end of *pssP2* was under control of the p*lac* promoter. The localization of the promoter ensured that the genes downstream of *pssP2* would be transcribed and the phenotype of the mutant would not be result of polar effects (**Figure 2C**).

**Figure 4:**
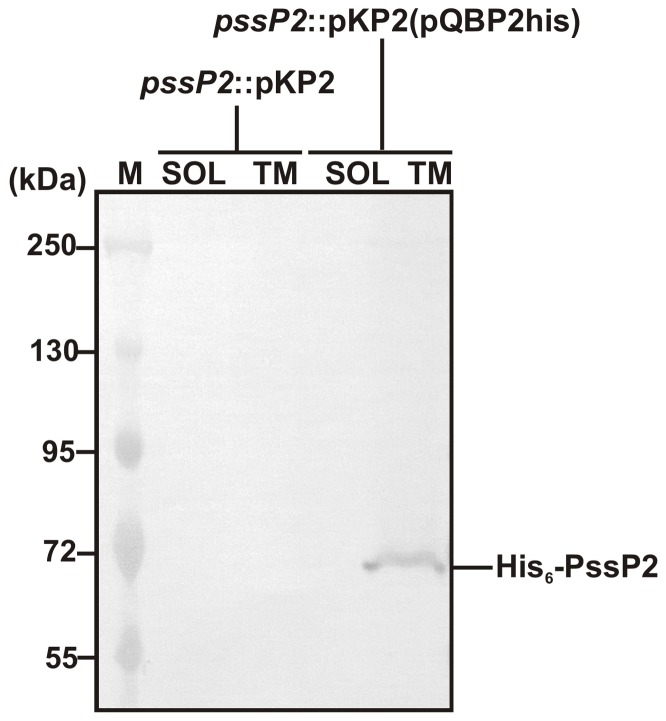
Western immunoblot analysis with anti-His antibodies of subcellular localisation of His_6_-PssP2 protein. Fractions analysed contained soluble proteins (SOL) and membrane proteins (TM) of the *pssP2*::pKP2 mutant and the complemented strain.

To complement the mutant phenotype, the *pssP2*-overexpressing plasmid pQBP2his was introduced into the *pssP2*::pKP2 strain. The expression plasmid pQBP2his ensured production of His_6_-PssP2 protein; however, the recombinant protein was poorly detectable in *E. coli*. A probable cause of that was the overrepresentation of arginine codons rarely used in *E. coli* (51% of all arginine codons in *pssP2*). The problem was previously encountered for other membrane proteins involved in polysaccharide synthesis, i.e. Wzy protein in *Shigella flexneri* [60] and PssL in RtTA1 [20]. Recombinant His_6_-PssP2 was easily detectable when the expression construct was introduced into the *pssP2::*pKP2. The protein detected in the cell lysate of *pssP2*::pKP2(pQBP2his) cells by Western blotting with anti-His antibodies had a molecular mass of ∼65 kDa (**Figure 4**). The presence of the protein was concomitantly checked in *pssP2*::pKP2(pQBP2his) cell fractions and His_6_-PssP2 appeared to be a membrane-embedded protein (**Figure 4**). Although *in silico* analyses suggested that PssP2 may be phosphorylated, Western blotting analysis with anti-phospho-Ser/Thr/Tyr antibodies gave no detectable signal, showing that PssP2 might not be phosphorylated, or at least phosphorylation is not detectable with the chosen method (data not shown).

**Figure 5:**
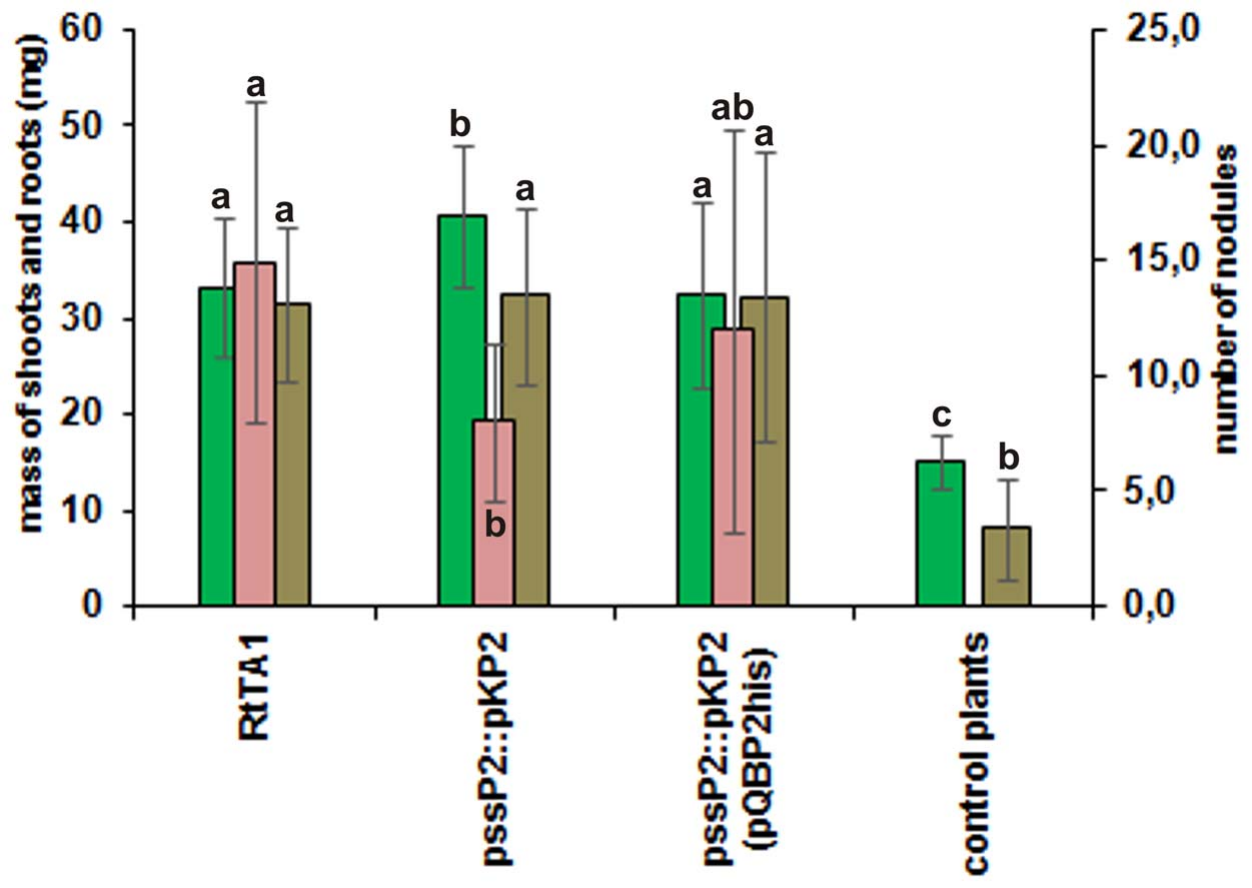
Symbiotic capabilities of the *pssP2*::pKP2 mutant and its complemented derivative compared with the wild type RtTA1 strain in a standard plant test. The mean values with standard error presented in the graph result from averaging the nodule number (*pink bars*), wet masses of plant shoots (mg/plant; *green bars*) and roots (mg/plant; *brown bars*) of 20 clover plants. The bars labeled with the same letters represent values which are not significantly different at p<0.05, while various letters represent values which are significantly different at p<0.05. Control plants were not inoculated with bacteria.

The *pssP2*::pKP2 mutant and its complemented derivative *pssP2*::pKP2(pQBP2his) were subjected to analysis of symbiotic efficiency and polysaccharide production. The *pssP2*::pKP2 mutant expressing a variant of the PssP2 protein lacking 153 aa from its C-terminus was symbiotically active, however the number of nodules formed on clover roots was significantly reduced in comparison to the wild type strain (**Figure 5**). The plants inoculated with the *pssP2*::pKP2 mutant gave a significantly better yield of shoot mass than those inoculated with the RtTA1 wild type and the complemented mutant (**Figure 5**).

**Figure 6:**
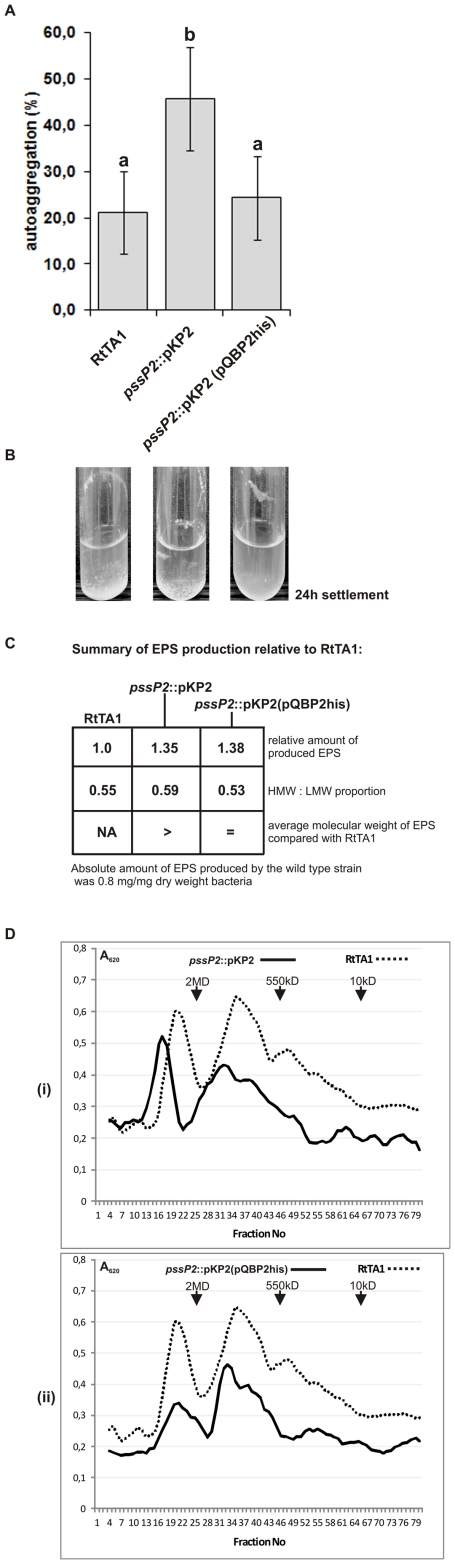
Exopolysaccharide production and autoaggregation properties of the *pssP2*::pKP2 mutant and its complemented derivative. A) Autoaggregation of RtTA1 wild type, *pssP2::*pKP2 mutant and the mutant carrying the pQBP2his plasmid as estimated quantitatively after growth and sedimentation in the 79CA medium. Autoaggregation is expressed in %; the higher the value the higher the autoaggregation. The bars labeled with the same letters represent values which are not significantly different at p<0.05, while various letters represent values which are significantly different at p<0.05. The extended bars represent standard error. B) Example photographs showing the autoaggregation of the studied rhizobia in 79CA liquid cultures (photographs were taken after the 24 hours of sedimentation). C) The table summarizes the EPS production by the studied strains. The amount of EPS produced is shown relative the amount produced by the wild type strain, which was 0.8 mg EPS/mg dry weight bacteria. D) Results of gel filtration chromatography of exopolysaccharides; the high-molecular-weight/low-molecular-weight (HMW/LMW) proportions were calculated as a ratio of peak areas: RtTA1 (dotted line in each graph), (i) *pssP2*::pKP2 mutant and (ii) *pssP2*::pKP2 mutant complemented with the pQBP2 plasmid. The retention times of dextran blue (2 MDa), dextran T550 and dextran T10 (10 kDa) molecular mass markers are indicated in each graph.

The *pssP2*::pKP2 mutant was affected in EPS production as it produced slightly more overall EPS than the wild type, but with an enrichment of LMW fractions (**Figure 6C**). However, the HMW fractions produced by the mutant had higher molecular masses than in RtTA1 (**Figure 6D-i**). The molar ratio of glucose/glucuronic acid/galactose in the EPSs from the RtTA1, the mutants and the complemented strain was 5∶2∶1, which is characteristic of EPS of *R. leguminosarum* bv. *trifolii* (data not shown).

It was shown that the quality and quantity of surface polysaccharides may influence autoaggregation of rhizobia [45], [61]. In line with this was the *pssP2::*pKP2 mutant ability to aggregate strongly in a liquid culture (**Figure 6A,B**). The ability to sediment at the bottom of tubes was significantly higher in the mutant strain (45.8%) than in the wild type (21.2%), and did not differ between the wild type and the complemented mutant (24.4%) (**Figure 6A**).

**Figure 7:**
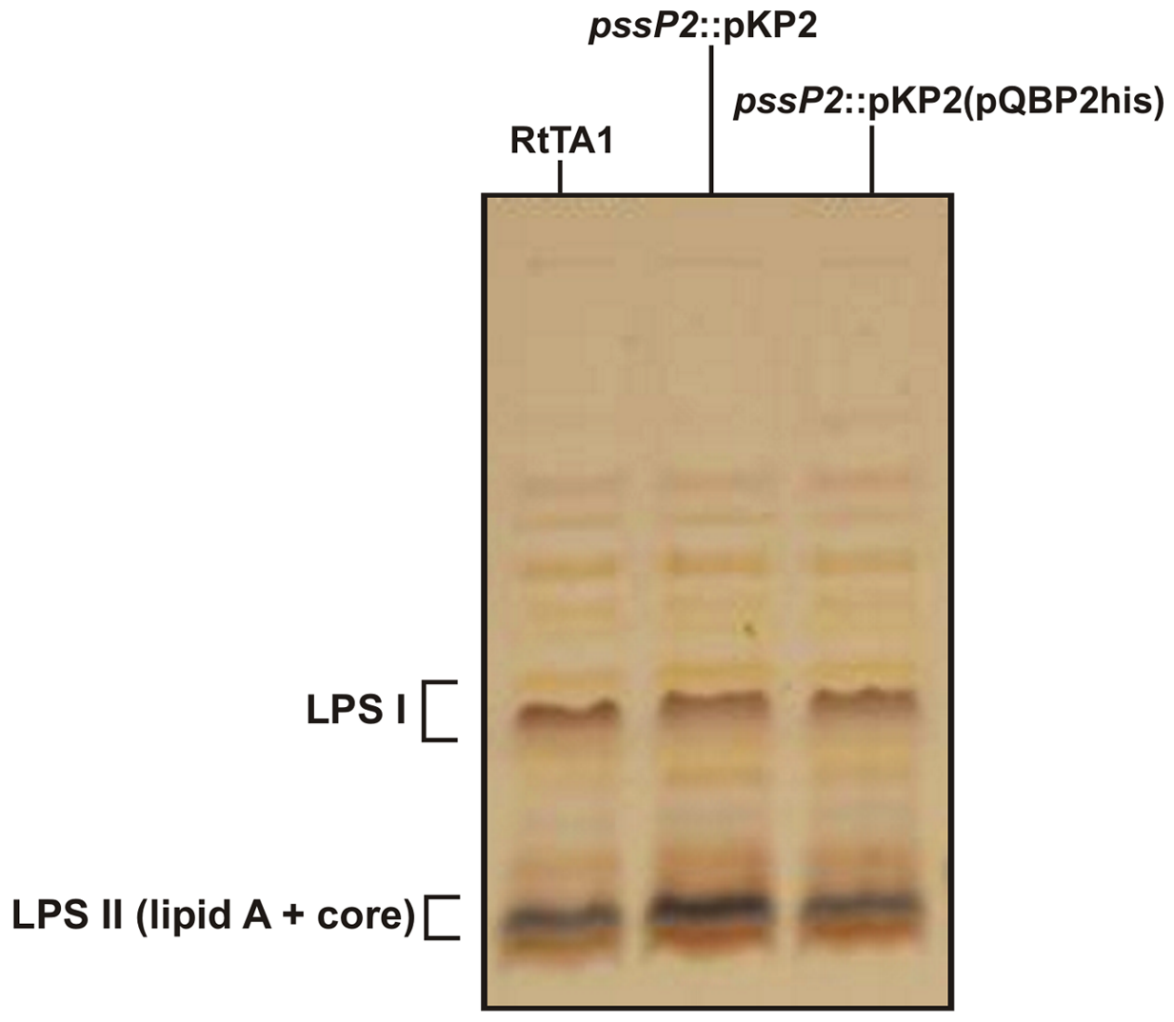
Silver-stained Tricine SDS-PAGE profiles of LPS from the wild type, the *pssP2*::pKP2 and *pssP2*::pKP2(pQBP2his) strains. LPS I, high-molecular-weight LPS which carries the O-antigen; LPS II, low-molecular-weight LPS, representing the core oligosaccharide and lipid A.

Production of the His-tagged PssP2 protein in mutant cells complemented its EPS phenotype (even without the induction of expression of *pssP2* from the vector), especially the masses of polymers and the autoaggregation ability. Moreover, overproduction of PssP2 in the mutant changed the proportion of HMW to LMW EPS slightly in favor of LMW fractions (**Figure 6C, 6D-ii**). The level of EPS production remained increased in the complemented strain (**Figure 6C**). Furthermore, the LPS of the *pssP2*::pKP2 mutant was equivalent to that of the wild type (**Figure 7**), providing further evidence for the role of PssP2 in EPS production.

### PssP2 interacts with PssT, PssP and PssC

**Figure 8:**
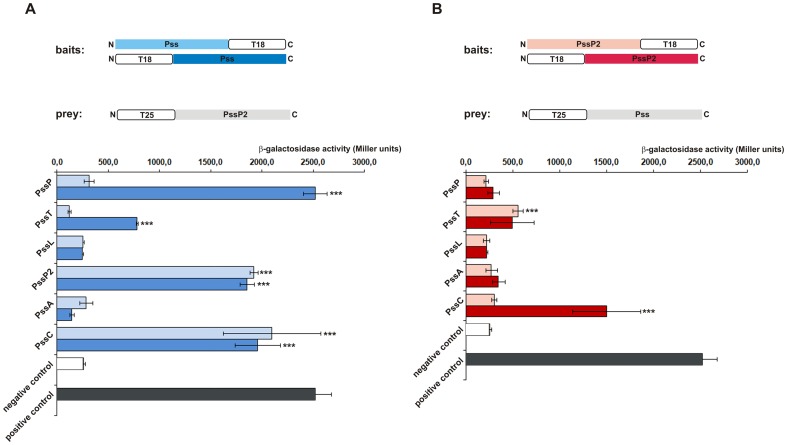
Interactions between PssP2 and other proteins involved in EPS production as analysed through the bacterial two-hybrid system. The graphs present the results of measurements of β-galactosidase activity in *E. coli* DHM1 carrying respective bait and prey plasmids, the combination of which are presented at the top of panels A and B. Each analysed protein, i.e. PssT, PssP, PssL, PssP2, PssA, and PssC, was encoded as a fusion protein with an adenylate cyclase fragment in vectors pUT18 (N-terminal fusions), pUT18C (C-terminal fusions) (baits), and pKT25 (C-terminal fusions) (preys) differentiated by colour. The β-galactosidase activity was measured in at least 3 independent assays for two colonies (biological repeats) in order to exclude clone-by-clone variation, averaged and expressed in U/mg of bacterial dry weight ± standard error. Positive and negative control values are presented at the bottom of each graph. The controls were: the two interacting leucine zipper domains expressed from pUT18Czip and pKT25zip (*positive control*), and T18 and T25 cyclase fragments in non-recombinant pUT18(pUT18C) and pKT25 (*the negative control*). The bars labeled with asterisks represent β-galactosidase activity values which are significantly different from the control at p<0.05.

In light of the changes in the quantity and quality of EPS in RtTA1 derivative producing shorter variant of the PssP2 protein, interaction analyses were undertaken for PssP2 and some of the thus characterized Pss proteins involved in EPS synthesis and transport. To this end, we employed a bacterial two-hybrid system [62]. Plasmids carrying *pssT*, *pssP*, and *pssL* genes were constructed previously [30]. In this work, plasmids encoding T18- or T25-fused *pssP2*, *pssA*, and *pssC* genes were constructed. The *E. coli* DHM1 *cya* reporter strain was sequentially transformed with all plasmids expressing fusion proteins. Positive clones representing interacting proteins (blue coloring of colonies and significantly higher levels of β-galactosidase activity than in the control) were obtained for the following pairs: PssP2-PssT (two combinations of fusion plasmids), PssP2-PssP (one combination), PssP2-PssP2 (both combinations; showing its ability to form homooligomers), and PssP2-PssC (three combinations) (**Figure 8**). The results obtained clearly showed that phenotypes resulting from *pssP2* mutation and overexpression might have come from the interrelation in which PssP2 is entangled, i.e. homooligomerization that is a characteristic property of polysaccharide co-polymerases and heterotypic interactions with the PssT polymerase, PssP co-polymerase, and at least one previously characterized glycosyltransferase encoded within the Pss-I gene cluster, PssC (**Figure 8**).

**Figure 9:**
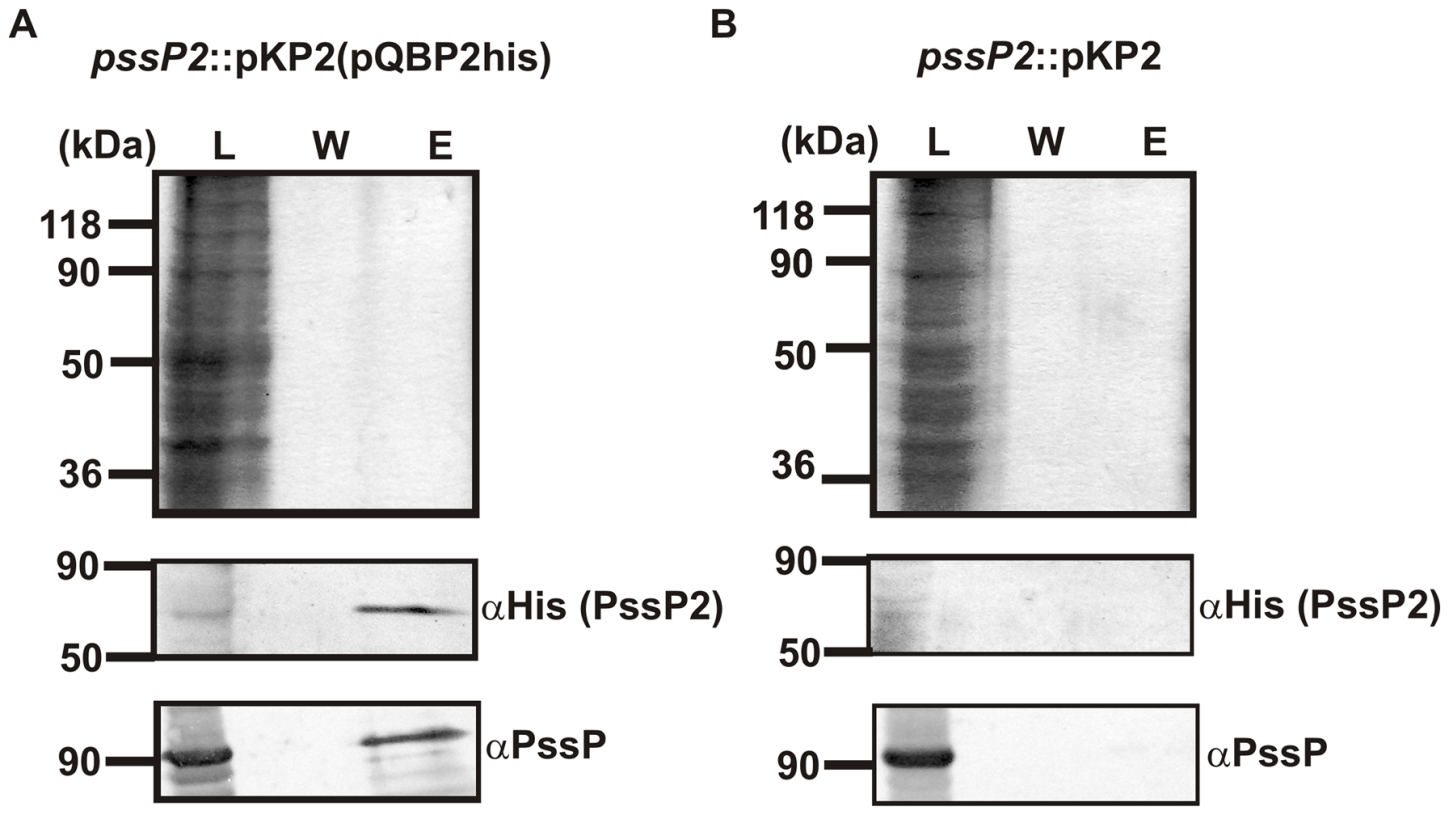
Analysis of the interaction between PssP2 and PssP in the *pssP2*::pKP2 mutant carrying the pQBP2his plasmid by a co-purification strategy. Samples of 40 µl of each fraction were separated by SDS-PAGE and visualized. (A) PssP is present in the fraction eluted from the resin, which equals to interaction between PssP and His_6_-PssP2, that was bound to affinity resin via a His-tag. (B) Negative control verifying that co-purification of PssP is dependent on its interaction with the His-tagged PssP2. L, material loaded to the resin; W, last wash (10 resin volumes); E, elution (1 resin volume). Western blots for each protein are shown below the corresponding gels.

The results obtained for the PssP2-PssP pair were significant only in one combination of fusion plasmids, i.e. pUT18C-PssP/pKT25-PssP2. To exclude a false positive result, a co-purification strategy was employed for *pssP2*::pKP2(pQBP2his) strain. In such background, PssP2 was His-tagged for purification through affinity chromatography, whereas the PssP remained untagged. Examination of the protein content of the fractions eluted from the affinity column after co-purification indicated that PssP2 and PssP interact with one another (**Figure 9A**). Control purification from cells not carrying the *pssP2*-expression plasmid demonstrated that PssP binding to the resin occurred through the interaction with a His-tagged PssP2 (**Figure 9B**).

## Discussion

Genetic control of EPS production in *R. leguminosarum* bv. *trifolii* was previously characterized at the molecular level and the functions of genes involved in the process were dissected. The PssT and PssP proteins encoded within the Pss-I region were shown to be involved in polymerization of EPS subunits and production of high- and low-molecular weight fractions of EPS (HMW and LMW) [21], [22]. Based on the results from the bacterial two-hybrid analysis, these proteins were proposed to interact with each other [30]. In the case of the PssP protein, it was shown that deletions in different domains caused its inability to form homooligomeric structures, but did not completely diminish the protein's property to interact with PssT [30]. Mutants with shorter PssP variants produced EPS, in which LMW fractions dominated [22]. In the case of PssT, deleting its C-terminal part made the protein more prone to homointeractions, but lack of the same domain made its interactions with PssP impossible [30]. Deleting the C-terminal part of PssT in the RtAH1 mutant resulted in production of EPS with prevalence of HMW fractions [21].

The results obtained in this work indicate functional interconnection between the PssP2 protein encoded within the Pss-II polysaccharide synthesis region with the EPS polymerization system encoded by the genes in the Pss-I region: glycosyltransferase PssC active at the EPS unit assembly step and proteins PssP and PssT involved in polymerization/transport outside the cell.

The mutant with a disrupted *pssP2* gene and encoding a protein lacking 153 amino acids from its C-terminal cytoplasmic domain produced more EPS than the wild type strain, and in addition to a quantitative increase, domination of HMW fractions containing chains with molecular masses higher than in the wild type was observed (**Figure 6C,D**). In line with this was the significant change in the autoaggregation properties of the mutant (**Figure 6A,B**). The *pssP2* integration mutant induced fewer, but all pink (and thus effective, nitrogen-fixing) nodules and the fresh masses of clover plant shoots were higher than in plants infected with the wild type (**Figure 5**). LMW EPS in *S. meliloti* was shown to be important for nodule invasion [63], [64], and HMW EPS is symbiotically inactive. It was shown that HMW EPS preserve *Rhizobium sullae* from desiccation [65]. The data concerning the role of HMW EPS in *R. leguminosarum* is scarce, however certain pieces of data indicate that it may be advantageous to rhizobia during the infection step. The phenotypes of *pssP2*::pKP2 (this work) and *pssT*::pAH1 [21] mutants support this idea. Both produce more HMW EPS, induce fewer but all effective nodules than the wild type, and the average green masses of plants inoculated with these strains is higher than for RtTA1 [21]. In other bacteria LMW and HMW polysaccharides play different roles in infection, virulence and persistence. In *S. flexneri*, the S-type Oag contribute to virulence [66], and VL-type Oag chains to bacterial resistance to complement [67]. In *Pseudomonas aeruginosa*, LPS with long (L)-type Oag chains contributes to greater resistance to complement and virulence in mice [68]. In *Salmonella typhimurium*, both L-type and VL-type Oag chains have been shown to confer resistance to complement [69], [70]. The S-type and L-type LPS Oag chains of *S. flexneri* confer colicin E2 resistance [71].

PssP2 was shown to interact with PssP, PssT, and one of the studied glycosyltransferases involved in synthesis of the octasaccharide EPS subunit, i.e. PssC, which acts by adding a glucuronosyl residue to the growing chain (**Figure 8**). Such a result underpins the hypothesis that PssP2 may function at the connection between EPS unit assembly and its polymerization and transport. PssP was shown to be indispensable for EPS production and null mutants produced no detectable amounts of EPS. Mutants with a shortened PssP protein produced more LMW EPS [22], and mutants with shortened PssT [21] or PssP2 – HMW EPS with higher molecular masses than in RtTA1. PssP2 overproduction in the mutant background led to a slight increase in the amount of LMW fractions than in the wild type. Taking into account the EPS phenotypes of *pssP*, *pssT*, and *pssP2* mutants, the fact that PssP did not interact with glycosyltransferases, PssP and PssP2 formed heterocomplexes, and both interacted with PssT, we can speculate that PssP2 and PssP may serve opposite roles in determining the extent of EPS polymerization. EPS produced by *Rhizobium* is generally characterized by the presence of LMW and HMW fractions. The involvement of two similar proteins in EPS polymerization would resemble involvement of the two Wzz proteins in the bimodal distribution of Oag in *S. flexneri*. In this case, two versions of the protein: chromosomally and plasmid-encoded are engaged. The chromosomal version is responsible for S-type Oag (short chains) [72] and the plasmid-encoded version of Wzz for VL-type Oag (very long chains) [73]. It was shown that these two Wzz proteins are differentially efficient and compete to control the degree of polymerization [74]. Moreover, in *S. meliloti* different paralogs of ExoP co-polymerase are involved in controlling the production of LMW and HMW EPS I under different physiological conditions [75].

Glycosyltransferases involved in synthesis of polysaccharides were shown to form a complex in the membrane [16]. It was proposed that the complex might interact with a flippase and a co-polymerase to regulate the length of produced chains [4]. One of the key players in such an interaction in the case of *R. leguminosarum* might be the priming glycosyltransferase PssA [19], as the interaction with the co-polymerase could regulate the flow of subunits to the polymerization centre. Following this, PssP might bridge PssL and PssT and be involved in HMW polymerization, while PssP2 could serve as a linker between glycosyltransferases and the polymerization centre, but being involved in LMW polymerization. Consequently, the phenotypes observed in the *pssP2::*pKP2 and its complemented derivative may reflect disturbances in the interactions between the components of the chain-length determination system composed of at least three components: PssP, PssT, and PssP2. It was shown for *Xanthomonas campestris* and *S. flexneri* that the level of proteins engaged in polymerization of EPS subunits or O-antigens, respectively, and their protein-protein interactions play an essential role in modulating the polymer chain length [74], [76].

The model in which PssP and PssP2 could serve opposite roles in determining the EPS length is further supported by their topologies. PssP was predicted to have four to five coiled coils. In the case of PssP2, only one periplasmic coiled-coil was predicted with high accuracy and one in the cytoplasmic domain, but with less confidence (**Figure 2**). The probability of coiled coil formation, location, and the number of the coiled-coil motifs is said to correlate with the degree of polymerization of the polysaccharides. If this were the case for PssP and PssP2, PssP would be responsible for HMW polymerization, while PssP2 for LMW polymerization. Moreover, the site of the mutation in PssP2 localizes near the secondary coiled-coil in the C-terminal domain of the protein (**Figure 2**). The C-terminal domain of PssP was not important for the interaction with PssT, but indispensable for homooligomerization [30]. If that had been the case for PssP2, the short variant of the protein in the mutant might have disturbed either homooligomerization or interactions with other proteins.

Contrary to an assembly model dependent on the stoichiometry of the complex members [77] are the results showing that the chain length determining function of PCP proteins depends on certain amino acid residues [78]. Previously, many mutagenesis studies on residues through Wzz proteins indicated that the function of modal chain length determination may be an overall property of the protein and may not be limited to one particular region [77], [79], [80]. It was reported that the Wzz level did not correlate with the length of O-antigen chains in *P. aeruginosa*. The amount of chains correlated with the level of protein production, but the length of O-antigen chains was dependent on a specific amino acid residue in a coiled coil domain [81]. Different amino acids may influence oligomerization and stability of the oligomers. Papadopoulos and Morona [82] noted that chain length was related to the stability of Wzz interactions; they described a positive correlation between dimer stability and the production of longer chain lengths. Changes in the oligomerization ability of mutated proteins may also be the case for the *pssP* and *pssP2* mutants. The PssP variants were not able to oligomerize and the mutants produced more LMW EPS [22], [30]. PssP2 also oligomerizes (**Figure 8**), thus secondary coiled-coils disrupted in the mutant might have affected its oligomerization/interaction properties. It seems reasonable that besides specific amino acid residues, any significant distortion of structures of Pss proteins may influence their interaction properties and thus the overall property of polymerization of EPS.

Several mutated Wzz proteins were undetectable via Western blotting but still produced a regulated chain length [82]. PCP proteins appear to be expressed at a higher level than Wzy polymerases, nevertheless still low. The promoter identified upstream *pssP2* is weak and the *pssP2* transcription is probably driven by a promoter preceding *pssY* with the medium activity comparable with the promoter of *pssP* [38]. *pssP2* lacks a strong RBS and has several rare codons in the 5′-end that may further support the low abundance of PssP2 in the RtTA1. It was shown that Wzz1 responsible for LMW polymers in *P. aeruginosa* could complement the phenotype even with the uninduced expression, while Wzz2 (HMW polymers) required induced expression for complementation [14]. The two proteins: PssP and PssP2 may have significantly different abundances in the cell, which would correlate with their functions and the possibly different mode of interaction with PssT. Data concerning promoter activity correlate with the phenotypes of *pssP2* and *pssP* mutants. The *pssP2*::pKP2 mutant was complemented via uninduced expression, which suggests that the level of protein produced without induction (**Figure 4**) was sufficient for the cell to restore the function. The *pssP* null mutant was not complemented and the reason for failure might have been the uninduced expression of *pssP* used for complementation [22].

The C-terminal cytoplasmic domain of PssP is characterized by the presence of an ATP-binding cassette domain. However, no tyrosine-rich motif, a hypothetic target for the phosphorylating/dephosphorylating activity, is present in this domain, excluding autophosphorylating activity similar to that of *S. meliloti* ExoP [83] and *E. coli* Wzc [84]. PssP2 is also similar to bacterial kinases involved in polysaccharide production, but it is devoid of any specific motifs and appeared not to be phosphorylated. Dissection of the functions of other genes in the Pss-II regions seems to be reasonable to clarify the functional importance of abundance of homologues implicated in polysaccharide synthesis. It cannot be excluded that the genes in the Pss-II region are important for modifications of the EPS HMW∶LMW ratio in the plant tissue or under unconsidered environmental conditions.

## Supporting Information

Figure S1 **Multiple sequence alignment between the PssP2 protein and the proteins mentioned in** **Table 2** **.** The order of sequences in the alignment is: PssP2 *R. leguminosarum* bv. *trifolii* TA1 (ABD36550) (marked with *red frame*), ExoP *Sinorhizobium meliloti* (P33698), Wzc *Escherichia coli* (P76387), CpsD *Streptococcus agalactiae* (Q3K0T0), Ptk *Acinetobacter johnsonii* (O52788), Etk *Escherichia coli* (P58764) and PssP *Rhizobium leguminosarum* bv. *trifolii* TA1 (ABD47316). Alignment was performed with the MAFFT tool and visualized with Alignment Viewer (http://toolkit.tuebingen.mpg.de/alnviz). Coloring of the alignment is based on the biochemical properties of the amino acids, thus the same color covers both identical and similar amino acids (if applicable).
(TIF)
