# Supplementary figures and images for: PssP2 Is a Polysaccharide Co-Polymerase Involved in Exopolysaccharide Chain-Length Determination in Rhizobium leguminosarum
Source: PLoS One. 2014 Sep 30;9(9):e109106. doi: 10.1371/journal.pone.0109106 (PMC4182512; doi:10.1371/journal.pone.0109106)

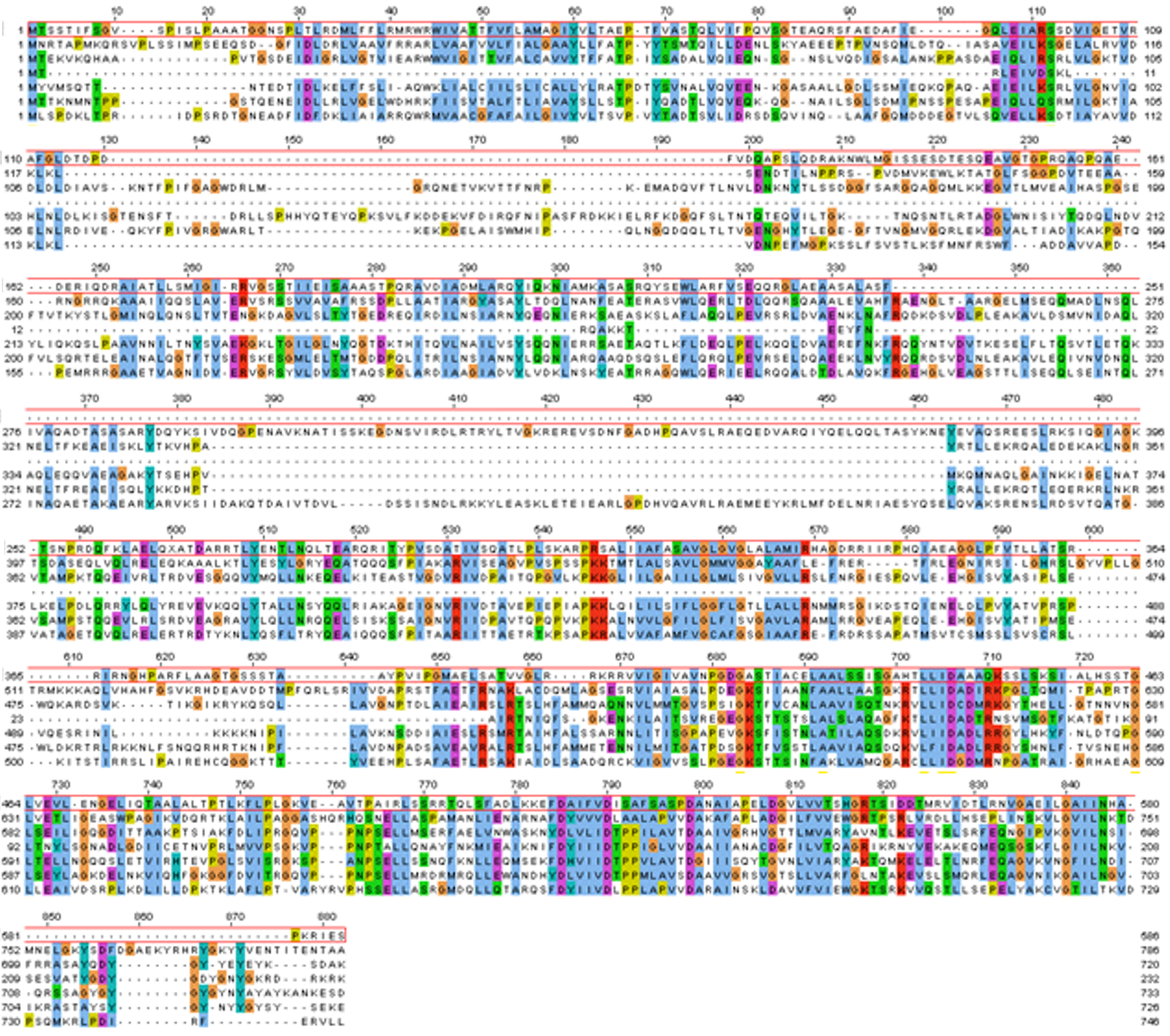

Supplement: Figure S1 — Multiple sequence alignment between the PssP2 protein and the proteins mentioned in Table 2 . The order of sequences in the alignment is: PssP2 R. leguminosarum bv. trifolii TA1 (ABD36550) (marked with red frame), ExoP Sinorhizobium meliloti (P33698), Wzc Escherichia coli (P76387), CpsD Streptococcus agalactiae (Q3K0T0), Ptk Acinetobacter johnsonii (O52788), Etk Escherichia coli (P58764) and PssP Rhizobium leguminosarum bv. trifolii TA1 (ABD47316). Alignment was performed with the MAFFT tool and visualized with Alignment Viewer (http://toolkit.tuebingen.mpg.de/alnviz). Coloring of the alignment is based on the biochemical properties of the amino acids, thus the same color covers both identical and similar amino acids (if applicable). (TIF) [file pone.0109106.s001.tif]
